# Immune and Inflammatory-Mediated Disorders: From Bench to Bedside

**DOI:** 10.1155/2018/7197931

**Published:** 2018-09-03

**Authors:** Marcella Reale, Lucia Conti, Diana Velluto

**Affiliations:** ^1^University of G. D'Annunzio, Chieti, Italy; ^2^Istituto Superiore di Sanità, Rome, Italy; ^3^University of Miami, Miami, FL, USA

Immune responses play a key role in maintaining tissue homeostasis, influencing nearly all organs and systems of the body including skin, gut, lungs, brain, and the cardiovascular system. Excessive or dysregulated immune responses and chronic inflammation represent a central driving force in many disorders, including infectious, inflammatory, and autoimmune diseases, as well as cancer. Cells of the mononuclear phagocyte lineage act as innate sentinels and are actively involved in regulating the balance between homeostasis and inflammation, thus ultimately contributing to the maintenance of the health condition. Age- and gender-related differences in immune response as well as in gut microbiota are emerging as important contributors in increasing the complexity in the diagnosis, treatment, and prevention of immune-mediated disorders.

In four original investigation articles, different autoimmune diseases were studied to find new biomarkers that could help explain the aetiology and pathogenesis of the diseases and be useful for new targeted therapy.

Myasthenia gravis (MG) is an antibody-mediated disease affecting the neuromuscular junction, caused by antibodies against the nicotinic acetylcholine receptor (AChR, AChR-Ab). Thanks to short half-life serum levels, free immunoglobulin light chains (FLCs) can be considered an instantaneous marker of B cell activity. In their study, U. Basile et al. showed an increase in free *k* chains in both AChR- and muscle-specific tyrosine kinase (MuSK-) MG while free *λ* chain levels were increased only in AChR-MG. Thus, they suggest that at least *k* chains can be considered a very sensitive circulating biomarker of B cell activation and humoral autoimmune response. This may represent a preliminary important study for a more detailed multicenter analysis.

It is clinically known that patients with one autoimmune disease tend to develop additional autoimmune diseases, and recently an increased prevalence of neuromyelitis optica (NMO) in patients with MG has been reported. To explain the exacerbation or increased susceptibility of patients with one autoimmune disease to developing an additional autoimmune syndrome, T. Mizrachi et al. established an animal model for both NMO and MG, using EAMG mice immunized with Torpedo AChR and then subjected to passive transfer of NMO-IgG or to immunization with AQP4-derived peptide. This study shows that injection of either AQP4 peptide or NMO-Ig to naïve mice caused increased fatigability and when the same molecules were injected into EAMG mice, the disease severity mediated by muscle weakness significantly increased.

In the course of primary Sjögren's syndrome (pSS), inflammatory cell infiltration consists mainly of lymphocytes infiltrating exocrine glands, which leads to their impaired function. The characteristic feature is generalized dryness. The disease develops slowly, and months can pass before a patient presents full spectrum of clinical symptoms. Insufficient treatment without inhibiting the autoimmune response leads to severe complications. A. Sebastian et al. attempted to answer the question whether it is possible to distinguish between patients with pSS and individuals with dryness caused by other pathologies without applying invasive diagnostic methods. The study included 68 patients with pSS and 43 healthy controls with dryness. They found that chronic fatigue syndrome is more common in pSS patients and can be a subjective distinguishing factor in the group of people with dryness.

E. Dziadkowiak et al. have planned their study to establish whether in patients with pSS without central nervous system (CNS) involvement, the function of the central portion of the sensory pathway can be challenged. The authors, by measuring somatosensory evoked potentials (SEP) to evaluate the function of afferent sensory pathways, confirmed dysfunction of the central sensory neuron, which indicates subclinical damage to the CNS in pSS patients.

Behcet's disease (BD) is an autoimmune and autoinflammatory disorder which origin is unknown, although both genetic and environmental factors play a role. Several genes have been found to be associated with the disease. Transcriptional profiling of PBCs, obtained from patients with active BD, to evaluate the role of the immune system in the pathogenesis of the disease was performed by A. Puccetti et al. The authors found up- and downregulated transcripts. By performing Gene Ontology analysis, they evidenced that most of the regulated transcripts can be related to inflammation, immune response, apoptosis, blood coagulation, vascular damage, and cell proliferation pathways, all playing a key role in BD.

The mechanisms contributing to the chronic inflammatory condition underlying some immunomediated disorders have been investigated or reviewed in a number of articles.

Lobular inflammation and mixed portal/periportal inflammation were observed in recurrent hepatitis C virus (HCV) infection and in acute cellular rejection (ACR), respectively. The aim of the research by A. I. Gomaa et al. was to evaluate whether the origin of macrophages and the immune mediator CXCR3 could help in differentiating between acute recurrent HCV infection and ACR after liver transplantation. Analyzing the expression of CD68 and CXCR3 in the postliver transplant biopsy in cases of recurrent HCV infection and cases of ACR, the authors found that CD68 was expressed in both recurrent HCV infection and ACR, and in patients suffering from recurrent HCV, stronger CD11b deposits in liver biopsies were also detected. On the other hand, CXCR3 is a marker and plays a considerable role in acute rejection following liver transplantation. The authors concluded that macrophages infiltrating the liver tissue after transplantation can distinguish between ACR by upregulation of CXCR3 and recurrent HCV infection by predominantly expressing CD11b.

H. Li et al. have reviewed the role of the innate immune system, inflammatory cells, immunoglobulins, immune-mediated mechanisms, and key cytokines in the pathogenesis of abdominal aortic aneurysm (AAA), a common degenerative cardiovascular disease. Reviewed studies demonstrate that immune-inflammatory reactions play a key role in AAA formation, development, and progression opening the door to the individuation of molecular targets and that, although a good deal of strategies have been proposed, the clinical practice is still lacking a valuable test.

G. Gelders et al. have reported current concepts of neuroinflammation and its involvement in Parkinson's disease- (PD-) associated neurodegeneration and interventions that could modify the pathological immune response in PD. In particular, the potential link among *α*-synuclein, activated microglia, increased expression of Toll-like receptors (TLRs), and several proinflammatory mediators, which consequently activate peripheral immune response, might open novel therapeutic options to modulate disease progression and outcome.

Among the recognized chronic inflammatory disorders is atherosclerosis, that represents a major threat to public health worldwide as is the main cause of cardiovascular diseases. Atherosclerosis is induced by oxidized low-density lipoprotein (ox-LDL) accumulation in the arterial intima under hypercholesterolemic conditions, which generates a state of chronic vascular inflammation. Multiple innate cell types contribute to this pathophysiological process, with macrophages playing a major role. In their original research paper, Y. Wu et al. reported, in the mouse macrophage model RAW264.7, that Toll/IL-1R domain-containing adaptor-inducing IFN-*β* (TRIF), a key adaptor of TLR3/TLR4-mediated signaling, plays an important role in regulating the ox-LDL-induced inflammatory response. They specifically demonstrated that TRIF modulates the expression of BIC/miR-155 and the downstream SOCS1-STAT3-NF-*κ*B signaling pathway via ERK1/2 activation, highlighting the potential role of TRIF as a novel therapeutic target for atherosclerosis. Y. Hao et al. investigated the mechanisms of the chronic inflammatory response contributing to visceral hyperalgesia (VH), providing new insight into the mechanisms involved in the antinociceptive effect of protease-activated receptor 4 (PAR4) activation. VH characterizes subjects with irritable bowel syndrome (IBS) who perceive excessive pain during abdominal distension. Activated colonic mucosal mast cells (MC) have been shown to play a crucial role in this process, through the release of proinflammatory mediators like tryptase, iNOS, IL-1*β*, and P2X7. The authors confirmed the expression of PAR4 in this cell type in a rat model of VH and its role in blocking the induction of proinflammatory mediators, suggesting that, in the gastrointestinal tract, the antinociceptive effects of PAR4 activation are mediated, directly or indirectly, by MC. They also hypothesized that nociceptive receptors could represent additional targets for modifying pain in gastrointestinal disorders such as IBS and inflammatory bowel diseases.

Myocarditis, mostly induced by viral infections, is an immune-mediated disorder resulting from both direct virus-triggered damage and indirect lesions induced by the host immune system. In particular, dilated cardiomyopathy, a complication of myocarditis, results from host immune response-induced killing of virus-infected and virus-uninfected cardiomyocytes and can lead to death. L. Zhao and Z. Fu summarized in their review article the specific role of host immunity (autoimmunity) in the development of viral myocarditis and in dilated cardiomyopathy.

Regarding cardiovascular diseases, in another review article, W. Zhou et al. reported on a novel mediator, the NLRP3. The NLRP is a subfamily of the nucleotide-binding and oligomerization domain- (NOD-) like receptors (NLRs) containing a pyrin domain. The NLRP mainly participates in inflammasome formation that is linked to many cardiovascular diseases. In this review, the authors presented the current knowledge about the function of NLRP in vascular disease, ischemic and nonischemic heart disease, and they discussed the potential therapeutic options targeting the NLRP3 inflammasome.

Human gut microbiota, as well as microbiota associated with other body sites (i.e., oral cavity, airways, and skin), is increasingly recognized for its important role in health. Microbiota and host mutually affect each other, and this intimate relationship strongly contributes to the maintenance of homeostasis. Disruption of this equilibrium has been associated with several chronic inflammatory diseases.

In this special issue, F. A. Salzano et al. have reviewed recent studies documenting the emerging role of nasal microbiota in reactive nasal inflammatory conditions, where the effects of allergens and environmental agents are mediated by host factors, including innate and adaptive immune responses. The critical role of nasal microbiota in coordinating these components and the contribution of microbial composition in affecting the onset and progression of allergic or nonallergic inflammation were discussed. Likewise, R. Yang et al. discussed the role of structural changes in gut microbiota composition in inducing immunological changes and in sustaining chronic hepatitis B virus infection and liver inflammation. The role of innate immunity components such as TLRs in linking intestinal flora and liver immunity is also reviewed.

Given the need of improving cancer prognosis, Y. Gidron et al. have studied the influence of the vagus nerve on tumorigenesis and observed that the vagus nerve may slow tumor progression because it inhibits inflammation. They have examined the relationship between a new vagal neuroimmunomodulation (NIM) index and survival in pancreatic cancer and in non-small-cell lung cancer. They found that the NIM index, reflecting vagal modulation of inflammation, may be a new independent prognostic biomarker in fatal cancers.

An interesting study on ventilator-induced lung injury (VILI) in preterm newborns has been also published in this issue. Here, the authors, C. Gutiérrez Carvalho et al., analyzed any association between the oxygen levels at blood sampling and plasma levels of the interleukins IL-6, IL-1*β*, IL-10, and IL-8 and TNF-*α* in preterm newborns under mechanical ventilation (MV) in their first two days. The study was conducted on 20 neonates (gestational age 32.2 ± 3 weeks) with severe respiratory distress. Blood samples were collected right before and 2 hours after invasive MV. The newborns were separated according to oxygen requirement: low-oxygen (≤30%) and high-oxygen (>30%) groups. In the high-oxygen group, IL-6, IL-8, and TNF-*α* plasma levels increased significantly after two hours under MV. Despite the small sample studied, data showed that there is some relationship between VILI, proinflammatory cytokines, and oxygen-induced lung injury, but a study considering oxidative marker measurements is needed. It seems that less oxygen may keep safer saturation targets playing a less harmful role.

Finally, a large amount of evidence has demonstrated that neuroinflammation plays a significant role in both acute and chronic neurodegenerative disorders including Parkinson's disease, Alzheimer's disease, multiple sclerosis, stroke, and traumatic brain injury. Y. Fu et al. reviewed the role of excessive microglial activation inducing inflammation-mediated neuronal damage and degeneration. They explored new herbal compounds that are able to suppress neurotoxicity via inhibiting microglial activation. The therapeutic targets and pharmacological mechanisms of these compounds have also been discussed in the review.

This special issue is a collection of original or review articles submitted by investigators representing eight countries across Europe, Asia, Africa, and South America, to highlight some of the objectives achieved in basic, translational, and clinical immunology. It provides a glimpse on some selected immune-mediated disorders highlighting the cell types and molecular mechanisms involved in the damage triggered by host immune responses either directly or following virus infections or changes in commensal flora composition.

